# Expression of DNA-PKcs and BRCA1 as prognostic indicators in nasopharyngeal carcinoma following intensity-modulated radiation therapy

**DOI:** 10.3892/ol.2013.1196

**Published:** 2013-02-18

**Authors:** JIAO YANG, XIMING XU, YANRONG HAO, JIAXIN CHEN, HEMING LU, JIAN QIN, LUXING PENG, BIAO CHEN

**Affiliations:** 1 Cancer Center, Renmin Hospital of Wuhan University, Wuhan 430060;; 2 Department of Radiotherapy, Clinical Cancer Center, People’s Hospital of Guangxi Autonomous Region, Nanning 530021, P.R. China

**Keywords:** head and neck cancer, DNA double-strand breaks, immunohistochemistry, radiotherapy

## Abstract

The mechanisms of radiation-induced effects in cancer mainly involve double-strand breaks (DSBs) which are important in maintaining the stability of genes. The DNA repair genes breast cancer 1 (BRCA1) and DNA-dependent protein kinase catalytic subunit (DNA-PKcs) are capable of maintaining genetic stability through two distinct and complementary repair mechanisms for DNA DSBs, known as repair-homologous recombination (HR) and non-homologous end joining (NHEJ). DNA-PKcs is a member of the phosphatidylinositol 3-kinase (PI3K) family. The PI3K/AKT cell signaling pathway is implicated in cell migration and invasion. The BRCA1 protein is implicated in multiple complex cellular processes that are related to chromosome sensitivity to mutagens. To determine the protein expression and clinical implications of DNA-PKcs and BRCA1 in nasopharyngeal carcinoma (NPC) and cancer progression, we evaluated its expression status by immunohistochemistry in 87 patients who received intensity-modulated radiation therapy (IMRT). In NPC, negative expression of DNA-PKcs was detected in 35 of the 87 (40.2%) cancer types and was significantly associated with poor patient survival (P<0.05). The overexpression of DNA-PKcs and BRCA1 also led to significantly improved distant metastasis-free survival compared with patients who did not overexpress both genes, although the expression level of BRCA1 and distant metastasis-free survival were not closely correlated. In addition, multivariate analysis indicated that DNA-PKcs status is a predictive marker of distant metastasis-free survival. In conclusion, lower expression of DNA-PKcs may be correlated with higher distant metastasis in patients with NPC. DNA-PKcs may be a predictive marker of distant metastasis after IMRT, independent of the classical prognostic marker. BRCA1 may additionally exert a synergistic effect to predict distant metastasis-free survival.

## Introduction

Radiotherapy forms a part of the front-line treatment regimen for nasopharyngeal carcinoma (NPC) due to the high sensitivity of NPC to radiation treatment (RT). Intensity-modulated radiation therapy (IMRT) has recently prevailed in clinical radiotherapy due to its lower radiation doses compared with other external radiation techniques. The mechanisms of radiation-induced effects in cancer mainly involve double-strand breaks (DSBs) which are important in maintaining the stability of genes. Distant metastases are common in NPC and are the major cause of treatment failure (1). Methods to predict distant metastases of NPC have yet to be developed. Therefore, the identification of prognostic factors that are useful for the prediction of distant metastases, particularly biological parameters, may allow the development of individualized strategies and, thus, improved treatment results.

DSBs are believed to be one of the most lethal forms of damage induced by DNA damaging agents (2). DNA DSBs are repaired by 2 distinct and complementary mechanisms, homologous recombination (HR) and non-homologous end-joining (NHEJ) (3). In the NHEJ pathway, one of the first enzymes to be attracted to DSBs is the Ku70/80 heterodimer. Upon binding to DNA, the DNA-Ku70/80 scaffold recruits a large 460-kDa serine/threonine kinase called the DNA-dependent protein kinase catalytic subunit (DNA-PKcs) (4,5). Followed by the recruitment of DNA-PKcs and the associated end-joining proteins, including Artemis, XRCC4 and DNA ligase IV (6), DNA-PK activity is stimulated via phosphorylation which enables the NHEJ reaction to proceed (7). Unrepaired DNA ends may contribute to the development of chromosomal translocations by acting as transposable elements (8). Although it remains to be demonstrated, DNA-PKcs has a great potential as a tumor-suppressor gene (9). In addtion, DNA-PKcs is a member of the phosphatidylinositol 3-kinase (PI3K) family. The PI3K/AKT pathway is involved in many cellular processes, including proliferation, survival, apoptosis, migration, invasion and cytoskeletal rearrangements.

The breast cancer 1 (BRCA1) gene was the first breast cancer susceptibility gene to be identified in 1990 (10). Classically, BRCA1 has been considered to be implicated as a modulator of response to DNA damage induced by chemotherapy and radiation therapy through HR (11,12). The N-terminal RING and C-terminal domains confer ubiquitinligase activity and specific phosphoprotein binding to BRCA1, respectively. Until recently, it was believed that mutations in the BRCA1 C-terminal (BRCT) domain led to reduced HR, resulting in genomic instability and, ultimately, the development of cancer (13). However, the mechanisms by which BRCA1 contributes to HR have thus far been little studied. A single study claimed that BRCA1 promoted single-stranded DNA (ssDNA) formation in response to ionizing radiation, with BRCA1 subsequently accumulating at the resulting ssDNA sites, possibly contributing to a later step in homology-dependent repair (14).

Due to distant metastasis occuring mainly in the 2–3 years after treatment in NPC, as well as it being the major cause of treatment failure, in the present study, we aimed to assess whether the expression of DNA-PKcs and BRCA1 may be used as prognostic markers of distant metastasis-free survival in patients with NPC who underwent IMRT. To the best of our knowledge, no previous studies have analyzed these two components together in correlation with therapy outcome and survival.

## Methods and materials

### Patients

Between May 2007 and February 2012, 87 untreated patients with NPC, who were due to receive IMRT at the People’s Hospital of Guangxi Autonomous Region (Nanning, China) were enrolled in this study. Patients with a history of other cancers or with distant metastasis were excluded. Of these, 87 cases had adequate source tissue available for immunohistochemical staining. The patients’ clinical characteristics are described in Table I. Patients with American Joint Committee on Cancer (AJCC) stage I–II were treated with IMRT alone and those with stage III–IVB with concurrent chemoradiotherapy (CRT). The tumors were staged according to the TNM classification as presented in the AJCC Cancer Staging manual (6th edition) (15). At 1 month after completion of IMRT, the IMRT response was evaluated by clinical examination, CT or MRI scan when required. After CRT treatment, if the patients still exhibited residual tumor activity, neck lymph node dissection surgery was necessary followed by adjuvant chemotherapy. The patients were followed for a period between 5 and 61 months (median, 26 months). The study was approved by the Appropriate Committees for Human Rights in Research in our hospital, and written informed consent was obtained from each patient.

### Immunohistochemical studies

Tumor biopsy specimens obtained from the 87 patients with NPC before treatment were analyzed by immunohistochemical methods. The expression of DNA-PKcs or BRCA1 was detected in formalin-fixed and paraffin-embedded tissues only after using a high-temperature antigen retrieval technique. Tissue sections (4 *μ*m) on poly-L-lysine-coated slides were treated for antigen retrieval by boiling in citrate buffer (pH 6.0) for 10–15 min. The sections were then incubated overnight at 4°C with monoclonal antibodies to DNA-PKcs or to BRCA1 (Beijing Biosynthesis Biotechnology Co., Ltd., Beijing, China). The streptavidinbiotin complex method with peroxidase conjugate was used for detection and the peroxidase reaction was developed using diaminobenzidine as the chromogen. The sections were counterstained with Mayer’s hematoxylin solution and mounted in a nonaqueous mounting medium. When ≥50% of the tumor cells in the biopsy specimen were immunopositive for DNA-PKcs or for BRCA1, the patient was classified in the DNA-PKcs(+) or BRCA1(+) groups, respectively. When <50% of tumor cells were immunopositive, including loss of expression, the patient was classified in the corresponding low expression group. Positive controls were provided by Beijing Biosynthesis Biotechnology Co., Ltd. Negative controls were obtained after omission of the primary antibody.

### Study design and statistical analysis

The primary endpoint of the study was the rate of distant metastasis-free survival. A drawback of the study was that the follow-up time was relatively short which subsequently led to further analysis with regard to the 5-year overall survival rate being required. Survival analysis was performed using the Kaplan-Meier method, and the curves were compared using the log-rank test. Cox regression for multivariate analysis was performed to identify the prognostic factors that influenced actuarial survival. Correlations with protein expression were assessed using the nonparametric Spearman correlation test. Differences in the distribution of patient characteristics were analyzed using the Chi-square test. P<0.05 was considered to indicate a statistically significant result. All statistical analyses were conducted using the SPSS 17.0 statistical software program (SPSS, IBM, Chicago, IL, USA).

## Results

### Clinical outcome

Twenty-five (28.7%) and 60 (69%) of the 87 patients achieved an initial complete response and partial response to therapy, respectively. Only 2 (2.3%) of the 87 achieved stable disease. Of the 87 cases, 2 (2.3%) occurred as locoregional failures at the 8th and 23rd months after treatment, respectively. Twenty-six (29.9%) patients had a distant metastasis and these patients accrued for a period of 4 to 57 months (median, 26 months). Distant metastases occurred in the liver in 12 patients, in the lung in 9 patients and in the bone in 5 patients.

### Immunostaining analysis

**Figure 1:**
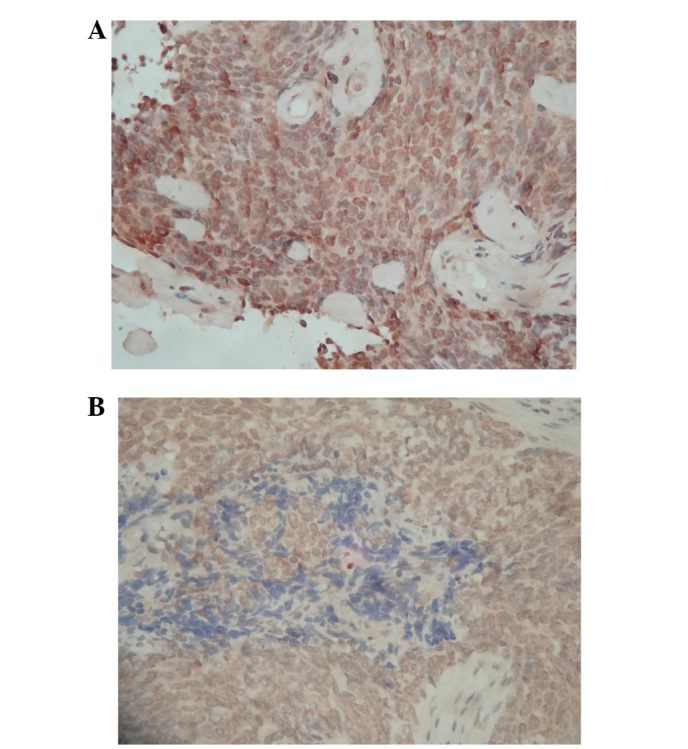
(A) Immunostaining for DNA-PKcs. (B) BRCA1 in representative cases from the high DNA-PKcs groups and high BRCA1 groups. Note the strong immunoreactivity predominantly in the nuclei of the tumor cells. DNA-PKcs, DNA-dependent protein kinase catalytic subunit; BRCA1, breast cancer 1.

The patients were divided into groups according to the expression level of DNA-PKcs and BRCA1 (high and low expression groups for each candidate marker). High expression levels of DNA-PKcs (Fig. 1A) and BRCA1 (Fig. 1B) were observed in paraffin-embedded biopsy specimens from 52 (59.8%) and 37 (42.5%), respectively, of 87 patients with non-disseminated NPC (Table II). There was no correlation between the expression level of DNA-PKcs or BRCA1 with age, gender, pathological subtype, T stage, AJCC stage or treatment.

### Correlation between clinical outcome and expression of DNA-PKcs and BRCA1

**Figure 2:**
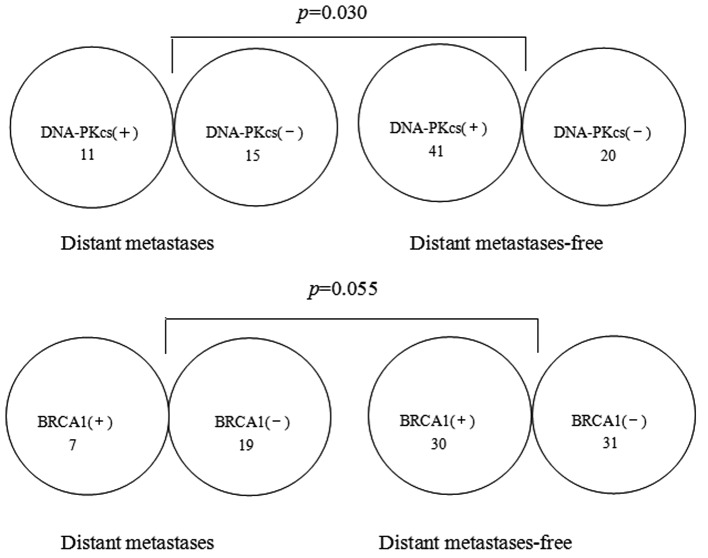
Correlation between the status of distant metastasis and the expression levels of DNA-PKcs or BRCA1 (P=0.030, P=0.055). DNA-PKcs, DNA-dependent protein kinase catalytic subunit; BRCA1, breast cancer 1.

**Figure 3:**
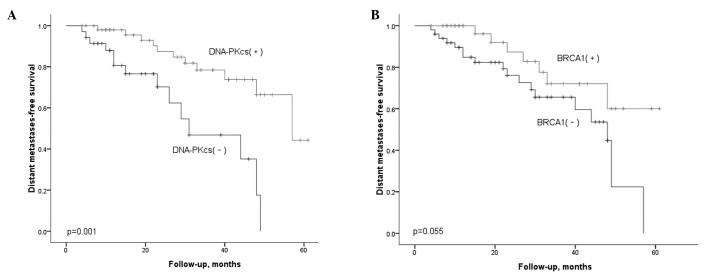
Survival times without distant metastases in patients with nasopharyngeal carcinomas. The upper and lower arm represent patients with DNA-PKcs(+) and DNA-PKcs(−), respectively. (B) Survival times without distant metastases in patients with nasopharyngeal carcinomas. The upper and lower arm represent patients with BRCA1(+) and BRCA1(−), respectively. DNA-PKcs, DNA-dependent protein kinase catalytic subunit; BRCA1, breast cancer 1.

**Figure 4:**
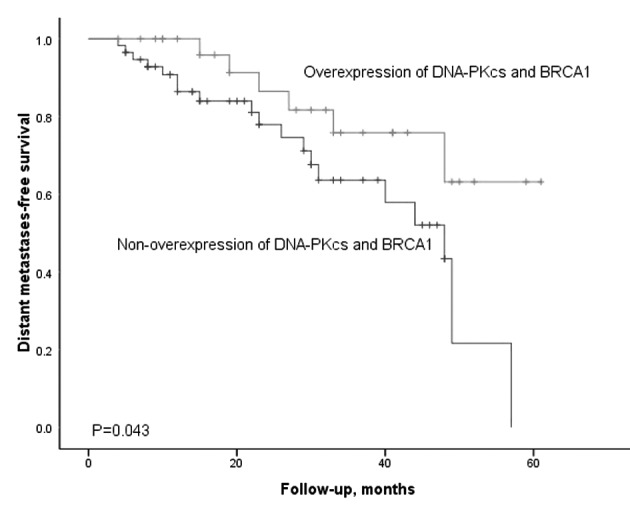
Survival times without distant metastases in patients with nasopharyngeal carcinoma. The upper and lower arm represent patients with overexpression of DNA-PKcs(+) and BRCA1(+) and the non-overexpression of them, respectively. DNA-PKcs, DNA-dependent protein kinase catalytic subunit; BRCA1, breast cancer 1.

Due to the improvement of radiation therapy technology, locoregional failure occurred in 2 (2.3%) of the 87 patients, which was too low to compare the differ ences between the DNA-PKcs and BRCA1 groups. There was a significant positive correlation between the expression level of DNA-PKcs and BRCA1 (r=0.374; P=0.001; Table II). Furthermore, a significant correlation was identified between the expression level of DNA-PKcs and distant metastasis-free rate (P=0.030; Fig. 2). Distant metastasis occurred in 15 (42.9%) of the 35 patients in the DNA-PKcs(−) group and in 11 (21.2%) of 52 patients in the DNA-PKcs(+) group. However, there was no significant correlation between the expression level of BRCA1 and distant metastasis-free rate. Distant metas tasis occurred in 19 (38.0%) of the 50 patients in the BRCA1(−) group and in 7 (18.9%) of the 37 patients in the BRCA1(+) group (P=0.055; Fig. 2). There was a weak correlation between the expression level of BRCA1 and distant metastasis rate, although this correlation was not statistically significant. If follow-up time was elongated and the number of patients were increased, the distant metastasis-free survival difference of the BRCA1 groups would be statistical significance. Figs. 3 and 4 illustrate the Kaplan-Meier estimated survival curves for distant metastasis-free survival in patients classified according to the levels of DNA-PKcs and BRCA1 expression in the tumor, respectively (P=0.001, P=0.055, P=0.001). We further performed a Cox regression for multivariate analysis (Table III). The expression levels of DNA-PKcs and AJCC staging demonstrated significant correlation with the distant metastasis-free survival. No significant differences were found between age, gender, ethnicity, pathological subtype or treatment in correlation with distant metastasis-free survival. However, no similar results were identified in the BRCA1 groups.

## Discussion

In our study, all of the patients received IMRT treatment. This is mainly since a larger volume of normal tissue is capable of being exposed to lower radiation doses with IMRT as compared with other external radiation techniques (16). NPC may frequently be cured by IMRT but metastasis and recurrence usually result in NPC treatment failure. Thus, prognostic tests for IMRT outcome based on biological markers are of particular interest to radiation oncologists. Therefore, we have examined the immunoreactivity of 2 molecules involved in our previous study (17) and also in the repair of damaged DNA. To the best of our knowledge, no studies have reported the predictive value of combining DNA-PKcs and BRCA1 protein expression in biopsy specimens for IMRT responsiveness in cases of NPC. Even single detection of the expression levels of BRCA1 in NPC has yet to be reported. We selected the immuno histochemical method using paraffin-embedded tumor tissues. This method is practical and efficient for clinical practice, since most cases of NPC are diagnosed only on the basis of the examination of small punch-biopsy specimens. If the immunoreactivity of these proteins are able to be used as *in vivo* indicators, specific treatment strategies may be designed for individual cases.

In the present study, numerous patients had stage III–IVB disease which led to low complete response rates, however, if the patients possessed residual tumor activity, neck lymph node dissection surgery was necessary followed by adjuvant chemotherapy. We first described the positive correlation between the expression levels of DNA-PKcs and BRCA1. This may be due to interaction between DNA-PKcs and BRCA1 which are capable of interacting to maintain genetic stability (3). Univariate analyses demonstrated that patients with a lower proportion of DNA-PKcs(−) tended to have a higher rate of distant metastasis than those with higher DNA-PKcs(+) in NPC (Fig. 3a). The improved distant metastasis-free survival in the patient group with high expression of DNA-PKcs was noteworthy, as we expected high-expressing tumors with an improved ability to carry out DNA DSB repair to be radioresistant. However, if the level of DNA-PKcs is low or even absent and p53 is mutated, cells may behave differently. Friesland *et al*(18) observed that p53^−^ (p53-negatove) and high DNA-PKcs levels were detected among patients with improved outcome, and the lowest survival rate was observed in patients with tumors that were p53^+^ (p53-positive) and had low DNA-PKcs expression which supported our hypothesis. We suggest that if both DNA-PKcs and wild-type p53 are present, the DNA damage is detected by DNA-PKcs and p53 activates an apoptotic response. If both the DNA-PKcs and p53 functions are defective, DNA damage is neither detected nor is p53-dependent apoptosis induced. If the level of DNA-PKcs is low or even absent and p53 is mutated, cells may behave differently. However, the exact mechanism of this phenomenon is unknown.

The close correlation between DNA-PKcs expression levels and metastasis may be due to it being a member of the PI3K family, including ataxia telangiectasia-mutated (ATM) and ATM- and Rad3-related (ATR) in response to DNA damage. The PI3K/AKT cell signaling pathway is implicated in cell migration and invasion (19). Our study also confirmed earlier studies by Lee *et al*(20,21), who reported that negative expression of DNA-PKcs in surgical specimens was significantly associated with tumor progression and poor patient survival rate in gastric cancer. In addition, DNA-PK activity may be determined by the transcription of the DNA-PKcs gene (22). Someya *et al* substantiate that the lower DNA-PK activity is correlated with chromosomal instability. Reduced DNA-PK activity profoundly affects the ability to repair DNA DSBs, resulting in the perpetuation of chromosome damage. Genetic instability correlated with low DNA-PK activity may cause a higher frequency of distant metastasis (23). Based on these observations, DNA-PKcs expression was thought to be a good candidate for a prognostic marker for NPC. To date, several studies support a central role for DNA-PKcs in radiation sensitivity (24,25). However, it is unclear whether radiation sensitivity was a predictor of the clinical outcome of an individual (26). Due to the application of IMRT, patients with radiation resistance were rarely observed in our study.

In the present study, no significant difference between the expression level of BRCA1 and clinical outcome was observed. BRCA1 is involved in a multitude of cellular functions, including HR and perhaps some forms of NHEJ. Until recently, it was believed that mutations in the BRCA1 C-terminal domain resulted in reduced HR, resulting in genomic instability and, ultimately, the development of cancer (13). As mentioned above, genetic instability may cause a higher frequency of distant metastasis. In the present study, there was a weak correlation between the expression level of BRCA1 and distant metastasis rate, although this correlation was not statistically significant. This may be due to different mutations in BRCA1 exerting different effects on the recognition and processing of DNA damage. In addition, the BRCA1 protein is implicated in numerous complex cellular processes that are correlated with chromosome sensitivity in mutagens. There are no other descriptions of BRCA1 immunoreactivity in NPC, so further studies will be necessary to compare with our results.

Univariate analyses demonstrated a statistically significant difference between the overexpression and non-overexpression groups (P=0.001, Fig. 4). Patients expressing a high level of DNA-PKcs and BRCA1 had an improved distant metastasis-free survival than those who were not. DNA-PKcs and BRCA1 are capable of interacting to maintain genetic stability. They are two key players in the NHEJ and HR pathway of DNA DSB repair, respectively. In our study, we confirmed that there was a significant positive correlation between the expression level of DNA-PKcs and BRCA1 (r=0.374, P=0.001). These factors supported our results that co-overexpression patients have a better distant metastasis-free survival despite their internal mechanism remaining unclear. To the best of our knowledge, this is the first report of the expression of BRCA1 and DNA-PKcs in paraffin-embedded NPC tumor tissues.

Finally, we identified that a high proportion of DNA-PKcs(+) cells and the AJCC stage were significant, independent predictors of patient distant metastasis-free after IMRT. In this study, age, gender, ethnicity, treatment, complete response rate, T stage and pathological type were not significantly associated with the probability of distant metastasis-free survival. Generally, AJCC stage is the most important prognostic factor for survival (1). The results of this study also indicated that the high proportion of DNA-PKcs-positive cells provide a strong molecular marker of improved distant metastasis-free survival in patients with NPC who are treated with IMRT.

In summary, cancer patients with lower DNA-PKcs tended to have the higher distant metastasis and the poorer prognosis. DNA-PKcs may thus be used as a marker to possibly predict the distant metastasis and poorer prognosis for patients with NPC. BRCA1 may have a synergistic effect with DNA-PKcs in predicting the distant metastasis and poorer prognosis. In the present study, we described the immunoreactivity of DNA-PKcs and BRCA1 in NPC for the first time. More studies will be necessary to confirm our results.

## Figures and Tables

**Table I:** Clinical findings of 87 patients with nasopharyngeal carcinoma.

Characteristics	Value	%
Gender		
Male	54	62
Female	33	38
Age (years)		
Median	46	
Range	17–76	
WHO pathology classification		
I (keratinizing)	0	0
II (nonkeratinizig)	7	8
III (undifferentiatd)	80	92
AJCC group (6th ed.)		
Stage I	0	0
Stage II	14	16
Stage III	35	40
Stage IVA or IVB	38	44
T stage		
T1	8	9
T2	27	31
T3	20	23
T4	32	37
CRT	73	83
RT alone	14	16

WHO, World Health Organization; AJCC, American Joint Committee on Cancer; CRT, concurrent chemoradiotherapy; RT, radiation therapy.

**Table II:** Immunohistochemical staining results for Ku70 and DNA-PKcs in nasopharyngeal carcinoma.

	DNA-PKcs(−)	DNA-PKcs(+)	Total
BRCA(−)	28	22	50
BRCA(+)	7	30	37
Total	35	52	87

Expression levels of DNA-PKcs and BRCA1 have a positive correlation (r=0.374, P=0.001). DNA-PKcs, DNA-dependent protein kinase catalytic subunit; BRCA1, breast cancer 1.

**Table III:** Cox regression prognosis analysis for distant metastasis-free survival in the DNA-PKcs groups.

Variate	B	SE	Wald	P-value	Exp(B)	95.0% CI for Exp(B)
Age	−0.003	0.023	0.014	0.906	0.997	0.953 to 1.044
Pathological subtype	0.442	0.661	0.449	0.503	1.557	0.427 to 5.680
AJCC stage	1.466	0.527	7.730	0.005	4.331	1.541 to 12.173
DNA-PKcs(+)	−1.593	0.461	11.966	0.001	0.203	0.082 to 0.501

DNA-PKcs, DNA-dependent protein kinase catalytic subunit; AJCC, American Joint Committee on Cancer. B, coefficient value; SE, standard error; Exp(B), relative risk; CI, confidence interval.
